# IABP: history-evolution-pathophysiology-indications: what we need to know

**DOI:** 10.1186/s13019-016-0513-0

**Published:** 2016-08-04

**Authors:** H. Parissis, V. Graham, S. Lampridis, M. Lau, G. Hooks, P. C. Mhandu

**Affiliations:** Cardiothoracics Department, Royal Victoria Hospital, Belfast, Northern Ireland

**Keywords:** Intra-aortic balloon pump, Mechanical circulatory support, Internal counterpulsation

## Abstract

Treatment with the intraaortic balloon pump (IABP) is the most common form of mechanical support for the failing heart. Augmentation of diastolic pressure during balloon inflation contributes to the coronary circulation and the presystolic deflation of the balloon reduces the resistance to systolic output. Consequently, the myocardial work is reduced. The overall effect of the IABP therapy is an increase in the myocardial oxygen supply/demand ratio and thus in endocardial viability.

This is an overall synopsis of what we need to know regarding IABP. Furthermore, this review article attempts to systematically delineate the pathophysiology linked with the hemodynamic consequences of IABP therapy. The authors also look at the future of the use of the balloon pump and conclude that the positive multi-systemic hemodynamic regulation during IABP treatment should further justify its use.

## Background

Different forms of support of the failing heart consist of Cardiopulmonary Bypass Pumps/ ECMOs, internal or external Counterpulsation and the various modes of Auxiliary heart pump.

Intraaortic balloon pump (IABP) is a form of internal counterpulsation, acting as an assisting circulatory support device. Diastolic augmentation during inflation potentially contributes to coronary, cerebral, and systemic circulation.

According to Freedman et al. [1] presystolic deflation lowers the impedance to systolic ejection. Myocardial work and oxygen demand are therefore reduced. The increase in cardiac output detected with intraaortic balloon treatment is between 0.5 and 1.0 l per minute.

Primarily the impact of IABP is to increase the myocardial oxygen supply demand ratio. The effectiveness of Intraaortic balloon counterpulsation on improving oxygen supply and subsequently left ventricular subendocardial blood flow would be best appreciated by examining the endocardial viability ratio [2] in a later chapter.

### Development of the idea of counterpulsation

The possibility to alter timing of pressure events during a heartbeat was conceived by Kantrovitz et al. [3]. External counterpulsatrion simulated when the hemidiaphragm wrapped around the distal portion of the thoracic aorta and stimulated during each diastole. The diastolic pressure was increased significantly as compared with control studies.

Simultaneously other researchers [4–6] experiment counter pulsation. They used femoral access to remove blood during systole and then replace it during diastole. Some haemodynamic impact was observed but the practical application of the technique failed to pursue.

Preliminary studies with an intraaortic balloon pump took place in 1961 by S. Moulopoulos and associates [7]. Latex tubing was tied around the end of a polyethylene catheter with multiple side holes. The distal end of the catheter was occluded so that the tubing could be inflated and deflated through the side holes in the catheter. The tubing, the catheter and the balloon formed a closed system that was filled with carbon dioxide; air pressure was applied intermittently to the tube in the cylinder and the carbon dioxide was expelled to inflate the balloon. The stroke was triggered with the aid of a timing circuit from the electrocardiogram (ECG) of the animal. The stroke length and the delay after the R wave of the electrocardiogram were preset so that the latex tubing was inflated during diastole and remained deflated during systole. Testing their balloon pump in a mock circulation and canine aorta concluded that it was possible to increase the diastolic blood flow in the arterial system and lower the end diastolic arterial pressure.

The concept of external counter pulsation was introduced by Dennis et al. [8] in 1963. This had become feasible by application of a G-suit (a diastolic leg compression method) covering over the lower extremity to mid abdomen, which sequentially inflated during diastole and deflated during systole, synchronous with the cardiac action.

The first clinical application of a successful treatment with IABP was reported in 1967. Intra aortic balloon pumping was advocated successfully in a 45 year old female who had sustained a myocardial infarction and was hypotensive, comatose and anuric in severe cardiogenic shock.

By the early 1970s and after a series of successfully treated patients with cardiogenic shock (The Kantrowitz-team had treated 30 patients) the IABP had been convincingly promised to be helpful in acute low cardiac output state following left ventricular failure.

Buckley et al. [9] looked at the hemodynamic benefit of the IABP and reported the results of treating the first eight patients in cardiogenic shock and confirmed that balloon inflation in diastole augments coronary perfusion and deflation just before systole markedly reduces resistance to the left ventricular ejection and thereby reduces cardiac work and myocardial oxygen consumption.

Mundth and co-workers [10] reported as early as 1970 a case of a patient who sustained cardiogenic shock following myocardial infarction who was stabilised with Intraaortic balloon counterpulsation; subsequently underwent coronary revascularisation and with the support of the balloon pump has had an uneventful recovery. This was the first report whereas the application of the intraaortic balloon pump extended successfully to support heart failure post coronary artery surgery.

Jacobey et al. [11] following the principles of counterpulsation in 1971 reported encouraging results for the treatment of cardiogenic shock in 18 patients with counterpulsation using an ascending aortic cannula.

In 1971 Feola and associates [12] looked upon the therapeutic impact of intraaortic balloon pump on a heart failure animal model. Three levels of successively worsening heart failure (by means of decrease in cardiac output and aortic pressure and elevation of the left ventricular end diastolic pressure) were reproduced. Institution of the IABP support followed by normalisation of left ventricular end diastolic pressure and left atrial pressure increased the stroke volume and ejection fraction as well as improvement of coronary and peripheral flow and so tissue perfusion. They concluded that intraaortic balloon pumping is an effective means of circulatory support providing that is being used early in the sequence of coronary occlusion -myocardial infarction -acute left ventricular failure. It is most effective in states of mild to moderate left ventricular failure. There was no effectiveness of the balloon pumping during the situation whereas cardiogenic shock with sustained hypotention for more than 30 min and 50 % drop in cardiac output, had been ensued.

Krakauer et al. [13] in the beginning of 1971 reported the experience with the intraaortic balloon in 30 cases treated for cardiogenic shock due to acute myocardial infarction refractory to conventional pharmacological treatment. They stated that counterpulsation could beneficially affect hemodynamic measurements. Ventricular end-diastolic pressure can be decreased, thereby reducing myocardial tension with a proportionate decrease in myocardial oxygen consumption. The coronary arterial perfusion pressure is increased and this together with reduced myocardial tension and decreased systolic ejection time permits enhancement of coronary blood flow. They concluded that the group of patients treated with balloon support early following the onset of the shock had a significant better prognosis.

In 1972 Bregman and coworkers [14] developed a dual chamber balloon consisting of a large proximal and a small distal balloon which inflated first; the idea was to block distal blood flow and augment flow proximally to the brain and coronary arteries.

The development of catheters and balloons of polyurethane material enable prolonged periods of counterpulsation.

In 1973 two different groups [15, 16] reported the successful utilization of IABP in patients who were unable to be weaned from cardiopulmonary bypass. Therefore, IABP support opened a new era in the perioperative management of patients with ventricular dysfunction during cardiac surgery.

By 1976 more than 5000 patients with post-cardiotomy low cardiac output had received IABP treatment in United States.

Continuous evolution brought a method of insertion of the balloon catheter percutaneously without the need for surgical cut down [17, 18]. Invasive Cardiologists then adopted this method; that transformed the entire field of IABP due to the aggressive expansion of indications for use of this device to different subsets of patients with advanced coronary artery disease unresponsive to medical management.

During the past decade major mechanical and engineering developments have allowed optimal timing of counterpulsation and on-line monitoring of blood pressure and cardiac output.

## The description of the balloon pump device

### The console

**Fig. 1:**
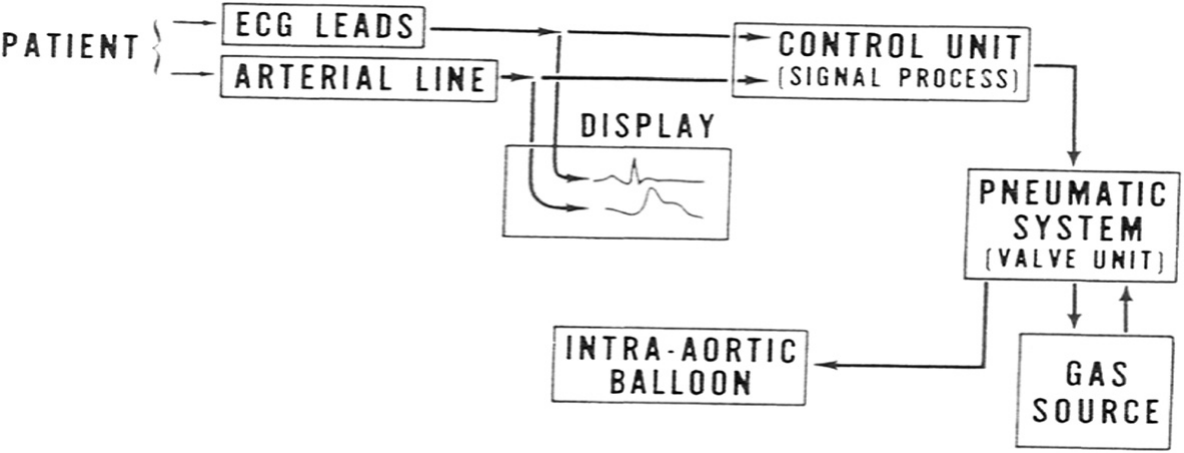
Description of the circuit between the patient and the intraaortic balloon pump (IABP). The IABP console includes the following: 1) A gas cylinder (usually helium, which has a theoretical advantage according to Hendrickx et al [14]; 2) a gas supply unit; 3) a monitoring system for recording the ECG and blood pressure; 4) a control unit that processes the ECG and generates a triggering signal. The latter unit is used for the timing of inflation and deflation of the balloon via activation of the valve unit, either opening the valve to supply gas or closing it to interrupt the gas flow

The IABP console delivers a specific volume of gas through a pneumatic system into a balloon during a predetermined time interval followed by retrieval of the gas, (Fig. 1). The console contains:

1. A cylinder of gas source (usually helium).
2. A valve unit, which allows delivery of the gas.
3. A monitor system for acquisition of electrocardiogram and arterial blood pressure.
4. Control unit which processes the electrocardiogram and develops a trigger signal; this is used for timing of balloon inflation and deflation by activating the valve unit and allowing either opening of the valve in order to deliver the gas, or closure of the valve unit in order to stop the gas flow.

### The driving gas

Both Helium and Carbon dioxide have been used as driving gases, however the use of helium has theoretical advantages according to Hendrickx et al. [19]: These include the speed of gas entry and retrieval as well as maintenance of a larger volume of gas within the balloon for a longer period of time; due to lower viscosity of helium as compared with CO2.

### Balloon catheters /sizing

#### Appropriate /inappropriate sizing/ balloon volume and aortic “occlusivity”

The size of the aorta is related to patient size, age and weight.

The ideal balloon for any patient has to have the length from the left Subclavian artery to the coeliac artery take off, the inflated diameter 90 to 95 % of that of the discending aorta and has to be equal in volume to the volume of blood in the aorta at any given time. The latter statement confirmed by Kantrowitz and colleaques who stated that the balloon is limited by the volume of blood contained within the aorta just prior to inflation (ie. The aortic volume doubles between a shock mean of 30 to 40 mm of Hg and a normal mean pressure of 80 to 90 mm of Hg). Further increases in pumping volume result only in distention of the aorta (due to aortic elasticity) and not in the effective pumping of the balloon.

IABC usage for adult patients has mainly been limited (82 % of the cases) to the use of 40 cc balloon, with membrane length (non tapered section plus tapered ends) varying between different manufacturer from 22 to 27.5 cm and inflated diameter between 15 to 18 mm, Table 1.

**Table 1:** Intraaortic balloon catheter sizes

Balloon volume	Balloon membrane length	Inflated diameter
25 cc	180 mm	13 mm
30 cc	230 mm	13.9 mm
34 cc	219 mm	14.7 mm
40 cc	260 mm	15 mm
50 cc	270 mm	18 mm

While in many clinical situations volume of 40 cc are appropriate, it should be said that too large IAB increases vascular morbidity whereas too small IAB reduces the cardiac benefit. 50 cc Balloon has been used in taller patients.

Diastolic augmentation is maximized when stroke volume is equal to balloon volume. If stroke volume is very low (ie 25–30 ml) or very high (95–100 ml) augmentation will be decreased.

#### Position of the balloon within the aorta

The closer the balloon is to the aortic valve, the greater the diastolic pressure elevation. It is obvious that local anatomical factors limit the position of the balloon within the aortic arch therefore the optimal balloon position will be that where the tip is situated distal to the left Subclavian artery take off. The proximal balloon end should be lying above the renal vessels. Incorrect balloon position results in reduced diastolic augmentation or vascular morbidity due to direct intimal injury or plaque distortion and embolization or finally direct occlusion of the arterial lumen.

### Hemodynamic criteria for mechanical circulatory support

Despite adequate preload, max pharmacological support and IAB pumping:

- Cardiac Index < 1.8lt/min
- Systolic arterial pressure < 90 mmHg
- LA or RA pressure > 20 mmHg
- UO < 20 ml/h
- SVR > 2100
- Metabolic acidosis

“Relative” Exclusion criteria

- Severe Peripheral Vascular disease
- Infection
- Hepatic disease
- Cancer with metastasis
- Severe Coagulopathy

### The balloon function

**Fig. 2:**
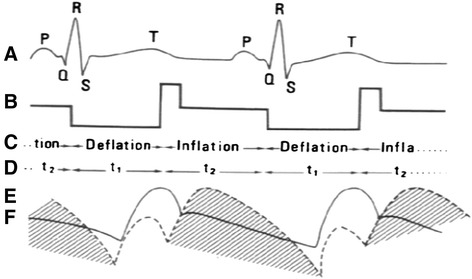
Haemodynamic function of the balloon. **a** ECG. **b**, **c** Balloon deflation, corresponding to systole during the cardiac cycle, and inflation, corresponding to diastole. **d** Balloon action. **e** Aortic pressure curve during balloon function. ** f** Aortic pressure

Following the principle of counterpulsation, the IAB is deflated during systole which coincides with QRS –T interval (R wave always triggers balloon deflation). In this manner balloon inflation during cardiac systole is prevented. The IAB is inflated during diastole, which coincides with T-P interval, Fig. 2.

## Cardiovascular consequences of IABP

Cardiovascular consequences are mainly due to the effect on preload and afterload [20]. Balloon inflation causes “volume displacement” resulting in a change in coronary circulation with redistribution of blood flow and alteration of oxygen consumption [21, 22].

Intraaortic balloon counterpulsation is instituted by insertion of a catheter mounted with a distensible polyurethane balloon in the patients descending aorta. Helium gas is shuttled from the balloon pump console. Inflation occurs immediately upon onset of diastole. Deflation occurs during isometric contraction. In a mechanical sense balloon inflation causes volume displacement. Total or regional blood flow is potentially improved with counterpulsation. Coronary circulation and perfusion to the aortic arch trifurcated vessels is potentially increased. Balloon inflation augments the intrinsic Windkessel effect, which augments peripheral perfusion [23]. At the time of deflation intraaortic blood volume is decreased with concomitant lowering of pressure. This process occurs at the end of diastole just as isovolumetric contraction is commencing and reduces the impedance against which the left ventricle must eject. Therefore decreasing afterload [24].

### Cardiovascular changes in systolic events

#### Decrease in systolic blood pressure

With proper timing if one compares the systolic pressures of non-assisted beats with systolic pressures following IABP assist one would conclude that intra-aortic balloon pumping results in a decline in systolic pressure by up to 10 % [25, 26]. A decrease in aortic systolic pressure in the course of balloon pumping indicates proper systolic unloading and afterload reduction. According to Norman et al. [27] an alteration in baroreceptor response may account for this effect.

#### Decline in pre-systolic (end–diastolic) aortic pressure

During IABP end diastolic aortic pressure is decreased up to 30 %; indicating systolic unloading.

#### Decrease of the isometric phase of left ventricular contraction

The Aortic valve during balloon pumping opens early therefore the isometric phase of LV contraction is decreased. This time interval is proportionally related to myocardial oxygen consumption [28].

#### Decrease in left ventricular wall tension and the rate of left ventricular pressure rise (dp/dt)

According to Urschel and collegues [29] the peak rate of LV pressure rise decreases with IABP up to 20 % compared with control values.

#### Effects in ejection fraction, cardiac output and starling law for the left ventricle

There is an increase in LV Ejection Fraction during IABP [30]. Likewise there is an increase in Cardiac Output between 0.5 and 1.0 Lt/per min or up to 30 % [31, 32].

The Starlings law curve for the left ventricle is affected by IABP [33]. There is a shift of the curve to the left showing improvement of the left ventricular function. This has been used as a prognostic indicator: by enlarged survivors show a sustained effect while non survivors show a shift to the right indicating ventricular deterioration.

### Cardiovascular changes in diastolic events

#### LVEDP and volume (preload)

The left ventricular diastolic volume is decreased due to systolic unloading.

The relation between left ventricular diastolic pressure change and left ventricular diastolic volume change (LV stiffness) exhibits a trend towards reduced values [34], a fact that translates into an improvement in left ventricular compliance.

### Cardiovascular changes in coronary blood flow

#### IABP and myocardial oxygen supply/demand

**Fig. 3:**
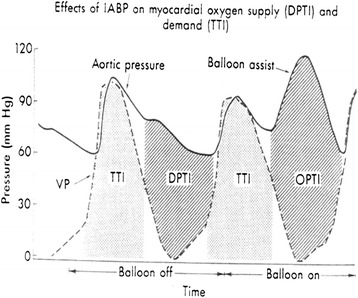
Effect of the intraaortic balloon pump on myocardial oxygen supply (DPTI) and demand (TTI). DPTI – diastolic pressure time index; TTI – tension time index

The IABP effect on improving myocardial oxygen supply could be better understood by examining the diastolic pressure time index (DPTI) and tension time index (TTI), Fig. 3.

DPTI reflects diastolic and subendocardial blood flow and depends on aortic diastolic pressure, left ventricular end diastolic pressure and diastolic duration. DPTI increases with IABP due to an increase in diastolic blood pressure and a decrease in end diastolic pressure.

The area under the left ventricular systolic pressure curve, which reflects myocardial oxygen demand, is termed tension time index (TTI). TTI decreases with balloon deflation due to a decrease in systolic blood pressure.

The ratio DPTI/TTI reflects the relation between oxygen supply and consumption of the myocardium representing the Endocardial Viability Ratio (EVR). A value of 1.0 or higher signifies a normal supply/demand balance. An EVR of less than 0.7 indicates severe myocardial ischaemia

Phillips et al. [35] introduced endocardial viability ratio (EVR) in conjunction with intra aortic balloon and stated that an increased EVR will reflect the increased blood flow occurring during augmentation, however only a period of EVR observation without balloon may assist in determining prognosis.

Bolooki et al. [36] states that with utilization of IABP the DPTI/TTI ratio is increased. The EVR index is useful as a criterion to decide early utilization of IABC in intra-operative failure.

It has to be clarified that TTI as an index of oxygen demand only accounts for pressure; on the other hand myocardial oxygen consumption is a function of force that is proportional to both pressure and volume, therefore although EVR will only reflect the decrease in pressure IABC produces a decrease in volume as well as pressure. As a consequence the decrease in myocardial oxygen consumption will be underestimated.

#### IABP and coronary artery perfusion

Balloon inflation displaces blood proximally increasing coronary perfusion by also increasing diastolic pressure and the diastolic perfusion gradient [37, 38].

There are various animal studies that assessed the contribution of the intra aortic balloon on myocardial perfusion. Results were variable. Kern et al. [39] stated that in animals with normal systemic arterial pressure intraaortic balloon pumping reduced myocardial oxygen consumption without significantly changing total coronary flow. In ischaemic animal models with low systemic arterial pressure myocardial oxygen consumption became dependent on coronary flow. IABP had little effect on perfusion to myocardial regions supplied by occluded coronary vessels.

Gewirtz et al. [40] undertook a study to determine if balloon pumping increased flow distal to a severe coronary stenosis, however found that despite using IABP blood flow distal to the stenosis remained unchanged.

McDonald et al. [41] found that IABC increased prestenotic but not postenotic flow.

However Folland and associates [42] contradicted this report. They observed relieved anginal symptoms in a population with coronary artery disease and concomitant severe aortic disease. They concluded that improvement in coronary flow must occur with pumping.

Freedman and associates [43] called myoconservation technique the initiation of IABC. He stated that the balloon action stimulated collateral circulation in the area surrounding the core of myocardial damage. Fuchs et al. [44] agreed that collateral circulation was encouraged during diastolic augmentation. Kern and associates [45] assessed intracoronary flow velocity during catheterization in 12 patients treated with IABC. Diastolic flow velocity time integral was recorded; the greatest increase in diastolic flow velocity time integral occurred in patients with a baseline systolic pressure of <90 mmHg. They concluded that IABC augments proximal coronary blood flow velocity by doubling the coronary flow velocity integral.

During counterpulsation aortic end diastolic pressure is lowered. Applying Laplaces low, lowering of aortic end diastolic pressure during static work (refers to development and maintenance of ventricular pressure before opening of the aortic valve) will decrease the amount of tension generated at the time the aortic valve opens. Therefore reducing myocardial oxygen consumption.

Akyurekli et al. [46] undertook a study to determine the systolic unloading effects of IABC independent of diastolic augmentation. This was carried out by counterpulsating dogs while their coronary arteries were perfused from an extracorporeal source. The perfusion pressure was lowered to produce acute cardiac failure. When IABC was instituted, systolic unloading (a reduction in left ventricular sustolic pressure) was evident in normotensive states, but not in hypotensive states (coronary perfusion pressure < 80 mmHg). During hypotension, aortic compliance increases which causes the aortic wall to expand with inflation of the balloon, therefore blood volume displacement does not occur. Moreover at the time of deflation, aortic pressure will not be lowered. Static work and myocardial oxygen consumption will therefore not be lowered.

#### IABC and peripheral blood flow

Peripheral blood flow is determined by pressure, resistance, length and viscosity. Balloon inflation during diastole increases the arterial pressure, which increases the arterial-venous gradient and thus improves flow. In addition balloon inflation in diastole displaces stroke volume and thus activation of the aortic baroreceptors inhibits the medullary vasoconstrictor reflex. Peripheral resistance decreases, which, as demonstrated by Poiseuilles low improves blood flow.

The impact of IABC on splachnic blood flow has also been studied. Landreneau et al. [47] studied the effects of intraaortic balloon pump assist upon splahnic blood flow during sustained hemorrhagic shock and following volume resuscitation. The IABP group was found to have a return to preshock splachnic visceral perfusion without the hyperemic reperfusion phenomenon seen in control animals. They concluded that IABPC during hemorrhagic shock appears to improve vasomotor control of splachnic blood flow by eliminating the hyperemic reperfusion phenomenon resulting in less reperfusion injury.

The effects of IABP upon postoperative renal function, was studied by Ahmed et al. [48]; they showed postoperative improvement in human renal perfusion during counterpulsation.

Swartz et al. [49] studied the influence of juxtarenal balloon position on renal blood flow. They found a 66 % mean decrease in flow while the IABP was in the renal position.

#### Weber and Janicki landmark paper

This paper is worth mentioned because it outlines the variables that influence diastolic pressure augmentation during balloon inflation [50]. The variables are:

1. Balloon position: The closer to the Aortic valve the greater the diastolic pressure elevation.
2. Balloon Volume: When the balloon volume is equal to the stroke volume the diastolic augmentation is maximized.
3. Balloon diameter and occlusivity: The greatest augmentation occurs with complete aortic occlusion.
4. Balloon Configuration/Driving gas & Timing.
5. Stroke volume: If stroke volume is less than 25 ml little diastolic augmentation can be expected.
6. Arterial pressure: The significance of aortic elasticity is illustrated by the fact that aortic volume doubles between a mean arterial pressure of 30 mmHg and a normal mean pressure of 90 mmHg. Our group [51] calculated an algorithm for optimal balloon sizing in order to improve diastolic augmentation and minimise patient-balloon mismatching.

## Indications

### Overall incidence of IABP utilization

The incidence of IABP treatment following Cardiac surgery is reported to be around 7 % in various units. This of course is dependent on the case mix and stratifying patients according to the Euroscore, one would obviously suggest that IABP usage correlates with ascending scoring.

According to various reports, Poor LV function, History of Myocardial Infarction, Female sex, Diabetes mellitus, Peripheral Vascular Disease and also Left Main Stem Disease are incremental risk factors for IABP utilization.

The unique physiological balance of benefits of IABP, include support of the coronary circulation [52, 53], as well as reduction in left ventricular stress and reduction in cardiac work-load [54].

The indications for use of an IABP involve two areas:

1. Temporary support of the left ventricular function due to cardiac failure, due to myocardial infarction or due to intraoperative injury.
2. Improvement in the oxygen supply/demand balance to decrease the extent of the ischaemic zone and to preserve myocardial viability.

However the efficacy of IABP is dependent upon the phase of myocardial ischaemia or the time elapsed from initiation of myocardial infarction as well as the stage of left ventricular function.

The value of the balloon pump as a circulatory assist device in the treatment of cardiogenic shock is well established [55–57]. Singh et al. [58] suggested criteria for which IABC would be most successful:

1. Triple Vessel disease with moderately preserved left ventricular function and good distal targets.
2. Significant mechanical lesions such as mitral insufficiency or ischaemic ventricular septal defect.

#### IABP following unstable angina refractory to medical measurements

Various clinicians must agree that during ischaemic episodes there is a potential window of opportunity were adequate hemodynamic support would ensure that adequate myocardium would remain viable to allow resumption of function, following coronary artery bypass grafting [59, 60]. This is the concept of myoconservation and where the intraaortic balloon pump exerts major impact.

Gold and associates [61] showed that the usage of IABP abolishes the pain, ameliorates ST segment elevation and prevents left ventricular tachyarrhytmia. The same group showed that [62] if the balloon treatment was followed with a CABG then outcome was statistically better than if the balloon treatment had not been instituted.

In unstable patients especially in the presence of left ventricular dysfunction, the use of IABP allows safe performance of diagnostic studies followed by surgical intervention. Zhang et al. [63] agree that following those lines of treatment, the operative mortality and the incidence of peri-operative infarction is less.

Langou and associates [64] report in 75 patients following the same regimen they had a 5.3 % operative mortality and a 6.6 % perioperative infarction rate. Furthermore a study of 55 patients with a similar presentation who were operated on without IABP, 14.5 % suffered operative death and 29 % had perioperative infarction.

Although the majority are not controlled non-matched series, they however still indicate that patients who are refractory to maximal medical therapy can be operated on with IABP stabilization with a low operative mortality and a low peri-operative infarction rate [65, 66]. Incremental risk factors for death include the subgroup of patients with poor left ventricular function, left main stem disease, left ventricular hypertrophy, unfavourable coronary anatomy, diabetic obese females and concomitant end-stage aortic valve disease.

#### IABP following myocardial infarction

In Theory, IABP could be used during acute myocardial infarction in order to decrease the size of the infarct, to support the cardiac function, to prevent infarct extension and to reduce complications associated with the event.

Miller and associates [67] published their clinical experience at St. Louis University with a series of 50 consecutive IABP patients. 33 patients (66 %) underwent IABP due to myocardial infarction. Chest pain was totally relieved in 29 patients (94 %) with improved hemodynamics in 46 of 50 patients (92 %). 19 patients (38 %) died, including 13 of the 33 patients admitted with acute myocardial infarction.

O Rourke et al. examined the effects of IABP on 26 patients with post acute myocardial infarction heart failure. The first group (*n* = 12 patients) experienced ischaemic pain. Ischaemic pain was not persistent in the second group (*n* = 14 patients). With institution of IABP more pronounced hemodynamic improvement exhibited in-group I.

Effects on ischaemic pain were impressive; pain was abolished within minutes for 11 patients and hours for one patient. Only one hospital death occurred in group I. Of the 14 patients in group II, 8 hospital deaths occurred.

The same group of investigators [68] in a randomised clinical study looked at the effects of IABP on post-myocardial infarction pump failure and showed no beneficial effects on definable end points. Therefore, in patients with acute infarction IABP is not employed except as a supportive measure to be followed by a myocardial revascularization procedure; when for example there is development of cardiogenic shock or if any of the mechanical complications following myocardial infarction have occurred. Once there are reasons for revascularization, IABP may be beneficial in decreasing the size of infarction and decreasing operative mortality [69].

In patients with acute myocardial infarction DeWood et al. [70] reported results with 40 patients treated with IABP for cardiogenic shock following myocardial infarction. Group I was treated with IABP and Group II with IABP and coronary artery bypass grafting. In hospital mortality between group I and II was 71 % versus 47 %. The portion of Group II that underwent treatment within 16 h from the onset of symptoms had a lower mortality (25 %) than the portion of Group II that underwent surgery more than 18 h after the onset of symptoms.(71 %).

Patients who have acute occlusion (coronary dissection or occlusion due to plaque rupture) of a major branch of the left coronary artery due to percutaneous intervention would benefit from IABP followed by emergency revascularization [71, 72].

#### IABP for ventricular arrythmias

In patients with acute myocardial ischaemia when tachyarrhythmia is refractory to second or third line antiarrhythmic therapy IABP treatment should be instituted [73] followed by cardiac catheterisation and coronary artery bypass grafting.

Almost all the ventricular dysrhythmias due to ischaemia are temporarily controllable with medication; therefore few patients would require IABP prior to a revascularization procedure. There is, however a role for using IABP in patients who have an unstable rhythm due to myocardial infarction early after myocardial revascularization.

Patients with ventricular aneurysms and arrhythmias with triple vessel disease amenable to bypass grafting have shown good survival results, however arrhythmia has persisted in 30 % of the cases [74] unless some form of aneurysm repair is carried out [75, 76].

#### IABP support for acute ischaemic mitral incompetence

Most frequently involves the posterior papillary muscle and the responsible coronary artery by 80 % is the right coronary artery. According to Chen et al. [77] early mortality is high in these patients (21 %).

IABP should be used in patients who have slipped into cardiogenic shock following post-infarction MR. This would allow haemodynamic stability in order to permit left ventricular catheter studies to be followed by mitral valve replacement. Concomitant bypass improves early and late survival [78, 79].

#### IABP support for acute ischaemic rapture of ventricular septum

In the majority of cases with cardiogenic shock pulmonary congestion ensues [80]. According to Khalid and colleagues [81] deterioration of the patients clinical condition is dependent upon the extent of involvement of the right ventricle and also the function of the left ventricle.

Poulsen et al. [82] reported on the extremely poor outcomes, with in-hospital mortality rates of about 45 % for surgically treated patients and 90 % for those treated medically.

IABP treatment during ischaemic rupture of the ventricular septum, increases the mean aortic pressure and cardiac output and decreases the right ventricular and pulmonary wedge pressure.

Patients should be operated upon as quickly as possible and a delay in surgery can lead to multi-systemic organ failure. These patients are at high risk for operative mortality and even with prompt surgical management, operative mortality remains significant because of heart failure and risk of fatal haemorrhage according to Sharma et al. [83].

Agnihotri and colleagues’ meta-analysis of 17 series from 1991 to 2009 reported five-year survival rates ranging from 33 % to 65 % especially if concomitant revascularization procedure had been advocated [84, 85].

#### IABP support during percutaneous coronary intervention (PCI)

The incidence of utilization of IABP in the course of cardiac catheterisation is reported by the International Benchmark Registry to be as high as 1 in 5 as an adjunct to high-risk PCI.

Various authorities around the world [86] have adopted a policy of “standby” IABP during angioplasty procedures in high-risk patients [87]. Treatment with balloon may be the most effective measure in the first few minutes following complicated angioplasty.

Relative criteria have been developed for selection of patients who should be considered for prophylactic IABP before PCI:

- Multi-vessel angioplasty in-patient with hypotension,
- Angioplasty of the only functional coronary artery
- Left main coronary artery angioplasty unprotected by a patent graft.

### Various rare indications for intra-aortic balloon pump

#### Bridge to cardiac transplantation

IABP mainly reduces the afterload thus improving the performance of the failing heart. In a series among 274 heart transplant patients, thirty-seven (28 %) required IABP as a bridge to transplantation.

#### High risk cardiac patients who are undergoing general surgical procedures

Two patients presented by Masaki et al. who underwent intraperitoneal surgery under the support of intraaortic balloon pump (IABP). In one patient, the IABP was inserted urgently because of the development of chest pain with significant ST depression on arrival in the operating room [88].

#### IABP support in paediatric population

The major problems encountered here are:

1. The small size of the aorta.
2. High elasticity of the aorta, which precludes effective balloon pumping.
3. Small stroke volume, which precludes balloon augmentation.

Pollack et al. [89] reported rather disappointing early experience with IABP support for heart failure following repair of congenital defects in infancy. However, recent advances in paediatric IABP technology have made its use feasible for children of all ages with acceptable morbidity. In selected groups of children with predominantly left ventricular failure, IABP has been an effective and lifesaving adjunct to conventional medical treatment of refractory low cardiac output. These developments are especially relevant to emerging countries where the cost of ECMO/VAD is prohibitively high; the use of IABP may be a more cost‐effective modality in appropriate patients [90].

#### Pulmonary artery balloon pumping

In theory would be indicated for patients with pulmonary hypertension such as after embolectomy or mitral valve replacement. Acute infarction involving the right ventricle would be a relative indication. Despite the obvious theoretical advantages, in reality the results are equivocal [91].

#### During sudden cardiac arrest

Combination of IABP with external compression during resuscitation theoretically improves coronary and cerebral perfusion.

#### Inability to be weaned off cardiopulmonary bypass/post cardiotomy cardiogenic shock

As early as the beginning of 1970s Berger et al. [92] and also Goldman and colleagues [93] realized that a major indication for use of an intra-aortic balloon pump is cardiac dysfunction with low cardiac output after heart surgery. The group of patients in whom IABP is deemed beneficial following unsuccessful separation off the cardiopulmonary bypass machine, include:

1. Patients with severe left ventricular muscle dysfunction and low ejection fraction who need an extensive operative procedure, which does not improve the cardiac performance immediately.
2. Patients requiring reoperations (redo-grafts) while they are suffering from an acute left ventricular dysfunction due to an unstable coronary syndrome.
3. When the ejection fraction in percent is lower than the end-diastolic pressure in mmHg.
4. Patients with severe long standing aortic stenosis and compromised ejection fraction especially when there is a need for associated procedures such as aortic root enlargement or coronary artery bypass grafting.
5. Patients with severe ischaemic mitral incompetence and dilated poor left ventricular function.
6. Patients with large left ventricular aneurysms and low ejection fraction.
7. Patients with left main stem coronary disease and an acute myocardial infarction in progress.
8. Post-operative right ventricular dysfunction.

Emergency use of IABP after cardiac surgery should be considered, when: all the causes of incomplete revascularization have been eliminated & there is a difficulty to wean from heart lung machine after attempting for 30 min at flow rates above 500 ml/min, with hypotension and low cardiac index despite increasing requirements of inotropic support (Dopamine > 10 mcg/Kg/min).

Utilization of IABP as an emergency measure for postoperative cardiac failure has consistently produced a survival rate of around 70 % by various groups such as Pennington et al. [94]. A 2010 study found a cumulative survival for a group of 2697 patients requiring IABP support post-operatively was 85.2 % at 4 years [95].

#### Brief summary of IABP complications

IABP complications are resulted during difficult insertion and malposition, prolong IABP stay and due to patient’s comorbidity such as peripheral vascular disease, small size patients, use of sheath of IABP and Diabetes.

A paper by Rastan et al. [96] identified IABP malposition to be a common finding on post insertion CT scans. Anatomic to balloon length mismatch was found in 68.2 % of the cases, with subsequently severe adverse effects.

Injuries resulting during IABP action could be overlooked unless catastrophic clinical implications are encountered; an important paper by Isner et al. [97] reviewed a total of 45 necropsy patients who have had an IABP inserted and who died within 105 days of the time of balloon insertion. Dissection of the aortoiliac axis occurred in nine patients and in none of them was the dissection suspected before death. In 4 out of those 9 patients, insertion occurred without resistance. In one out of the 3 patients that they had developed arterial perforation no complication of balloon insertion had been developed. In 2 out of the 3 patients that they had developed thrombosis intravascularly no clinical suspicion rose prior to death. Clinically silent arterial emboli occurred in 3 patients. They concluded that out of the 20 complications (in 16 patients) only 4 (20 %) had been suspected before death.

By enlarge, complications are reported to be primarily associated with the insertion process and prolonged balloon pumping, rather than removal or post removal monitoring.

Obviously, due to the nature of the IABP, the main complications relate to vascular injury, with studies suggesting vascular ischaemic complications of between 8–18 % with major limb ischaemia reported to be less than 1 % [98–102].

In a study published by our group [103], cold pulse-less foot was detected in 29.5 % of the cases. The ischemia resolved either with removal of the balloon (*n* = 18 pt) or with thrombectomy (*n* = 8 pt, 5.8 %). One patient developed gangrene and required amputation.

Thrombocytopenia, defined as platelets <150,000/mL or >50 % decrease from baseline, occurred in 57.9 % of patients.

Among patients undergoing IABP, thrombocytopenia is generally mild, appears to be unrelated to concomitant heparin use, and is not associated with an increased risk of major bleeding or in-hospital death [104, 105].

Rupture of the IABP is rare but can cause gas embolism and potential entrapment of the balloon within the arterial tree. This was first reported by Rajani et al. [106] however it is very rare, possibly less than 0.5 %. The proposed mechanism involves mechanical disruption of the balloon against an atherosclerotic plaque or extensively calcified aortic wall with resultant perforation and the negative pressure created during deflation traps blood within the balloon. The blood rapidly reacts with the helium causing a hard clot formation, which together with the tortuous atherosclerotic aortic environment results in entrapment of a semi-deflated balloon [107].

Finally, Complications of thrombosis and infection are related to the duration of IABP therapy while the limb ischemic problems are more a function of the atherosclerotic status of the common femoral artery and either the ratio of balloon catheter diameter to arterial lumen, or the difficulty of dealing with a severely atherosclerotic artery with loose plaques or fragility requiring excessive surgical manipulation.

## Conclusions

To summarise, the use of the IABP is justified because an increase in diastolic pressure during balloon inflation augments the coronary circulation. Further, pre-systolic deflation of the balloon reduces the resistance to systolic output; thusa decrease in myocardial work.

The efficacy of the IABP is reflected by the positive outcomes of the high number of patients who are weaned from the device. The success rate is higher for patients, in whom the device is deployed early, reflecting the reversible nature of ischaemic pathology and the positive contribution of the IABP to preventing the cascade of complications of ischaemic heart disease.

Mechanical assistance to the circulation is a rapidly developing field. The concept of intervention within the cardiac cycle in order to improve cardiac performance has been studied from every angle. Diastolic counterpulsation by means of IABP is the direct application however various manipulations in the systolic phase have been suggested by different researchers.

### Midsystolic counterpulsation

A Spherical balloon introduced into the ascending aorta with the diameter equal to that of the aorta and inflated for 80 msec during the middle third of the ejection phase with main effect to increase the coronary flow.

### End systolic counterpulsation

A small balloon introduced into the LV and inflated for 100 msec during late systole, thus reducing LV residual volume after cardiac systole and increases LV capacity for the next contraction. The result may be an increase in contractility and stroke volume. By inflating the balloon at the end of diastole the LV end diastolic pressure increases resulting in improvement of the stroke volume.

Future developments most likely would involve better definition of the subgroup of patients requiring IABP support, clear criteria for weaning and improvement in catheter technology and in drive consoles.

## Abbreviations

DPTI, diastolic pressure time index; ECMO, extracorporeal membrane oxygenation; EVR, endocardial viability ratio; IABC, intra-aortic balloon counterpulsation; IABP, intra-aortic balloon pump; LVEDP, left ventricle end-diastolic pressure; PCI, percutaneous coronary intervention; TTI, tension time index; VAD, ventricular assisted device
